# Aquatic macroinvertebrate diversity in mosquito larval habitats in São Tomé and Príncipe

**DOI:** 10.1371/journal.pone.0339486

**Published:** 2026-01-06

**Authors:** Maria Júlia Maciel Corrêa, Madizalda Ceita, Robert E. Ditter, Melina Campos, Hester Weaving, Andrew Goffinet, Claire M. Egan, João Viegas, Anthony J. Cornel, Gregory C. Lanzaro, João Pinto

**Affiliations:** 1 University of California Malaria Initiative, University of California Davis, Davis, California, United States of America; 2 Universidade de São Tomé e Príncipe, São Tomé, São Tomé e Príncipe; 3 Department of Pathology, Microbiology and Immunology, Vector Genetics Laboratory, University of California Davis, Davis, California, United States of America; 4 Department of Biological Sciences, State University of New York at Buffalo, Buffalo, New York, United States of America; 5 Programa Nacional de Eliminação do Paludismo, Centro Nacional de Endemias, São Tomé, São Tomé e Príncipe; 6 Department of Entomology and Nematology, Mosquito Control Research Laboratory, University of California, Parlier, California, United States of America; 7 Global Health and Tropical Medicine, LA-REAL, Instituto de Higiene e Medicina Tropical, Universidade Nova de Lisboa, Lisboa, Portugal; University of Mpumalanga, SOUTH AFRICA

## Abstract

Oceanic islands harbor unique aquatic ecosystems characterized by distinct macroinvertebrate communities that play vital roles in ecosystem functioning and stability. São Tomé and Príncipe islands (STP), located in the Gulf of Guinea, represent a model system where the primary malaria vector, *Anopheles coluzzii*, shares larval habitats with a diversity of aquatic taxa. Here, we evaluate macroinvertebrate diversity in permanent and temporary larval habitats typical of *An. coluzzii* in STP during the wet and dry seasons. We collected 5,208 macroinvertebrates belonging to eight classes, 15 orders, and 51 families. These included insects, crustaceans, spiders, annelid worms, springtails, and mollusks, with insects and crustaceans dominating collections. Diversity remained stable across the wet and dry seasons, but higher diversity was found in permanent habitats when compared to temporary habitats. We found 9 families (12% relative abundance) that included potential predators of mosquito vector larvae. Our results demonstrate that larval habitats of *An. coluzzii* support a dynamic community of aquatic macroinvertebrates. Establishing this ecological baseline is crucial for future assessments of community composition and for informing sustainable vector control management and biodiversity conservation on these islands.

## Introduction

Oceanic islands often have communities that are ecologically simpler than mainland systems but evolutionarily distinct. These communities are shaped by geographic isolation, limited colonization events, and unique adaptive trajectories [1,2]. Such features make islands valuable natural laboratories for studying aquatic communities and ecological interactions [1–3].

Tropical aquatic ecosystems on islands often harbor diverse macroinvertebrate communities that play crucial roles in maintaining ecosystem functioning [4]. Such ecological interactions encompass a broad range of relationships, including trophic interactions such as predation, as well as non-trophic interactions like competition and facilitation, all of which contribute to resource partitioning and community structure [5,6].

Despite their relatively reduced species richness compared to continental systems, these insular aquatic habitats are often characterized by specialization and unique species interactions that contribute to ecosystem stability [1,7,8]. Their diversity and composition reflect both local habitat conditions and broader regional processes [9]. Moreover, they provide a valuable basis for comparing communities across habitats, making them essential for understanding and conserving island biodiversity amid environmental changes [7,9], as well as for establishing ecological baselines [10].

São Tomé and Príncipe islands (STP), located in the Gulf of Guinea, exemplify these insular patterns [8]. Compared to mainland Africa, the freshwater ecosystems of STP host fewer species but exhibit higher levels of specialization, with interactions often magnified by the reduced complexity of insular food webs [11,12].

The rich endemic biodiversity of STP is well-documented for mammals, birds, and amphibians, but arthropods, especially aquatic macroinvertebrates, remain relatively understudied [13]. Macroinvertebrate biodiversity plays a vital role in ecosystem functioning, not only by contributing to nutrient cycling and food web stability but also by providing biological control of pest and disease vector species [14,15]. These aquatic systems include typical larval habitats of *Anopheles coluzzii*, the sole primary malaria vector species on the islands. The larval and pupal stages of *Anopheles* mosquitoes share aquatic habitats and interact closely with other organisms in the ecosystem and serve as prey for a diverse variety of predators [13].

Over the past five years, malaria incidence has risen across the African continent, reaching an estimated 265 million cases in 2023 [16]. STP has not been exempt from this trend, with cases increasing despite sustained control measures [16,17]. Features such as small size, reduced biological complexity, and the presence of a single malaria vector species, *An. coluzzii*, make the islands a promising setting for the deployment of a gene-drive genetically engineered mosquito (GEM) for malaria control [18]. However, real-world application of this innovative strategy requires extensive testing to evaluate potential ecological impacts, particularly interactions between GEM and non-target organisms [19]. Characterizing biodiversity and community structure in these systems is therefore a critical component of the environmental risk assessment framework, ensuring that such interventions align with principles of ecological safety and long-term sustainability [10,20,21–23].

This study aimed to characterize the biodiversity of aquatic macroinvertebrates inhabiting larval habitats in STP. The specific objectives were to: (i) document the aquatic biodiversity present in typical larval habitats of *An. coluzzii*; (ii) compare biodiversity patterns between temporary and permanent habitats, across wet and dry seasons, and between years; and (iii) identify potential predators of mosquito larvae. This baseline provides essential information on the structure of aquatic communities in larval habitats where *An. coluzzii* occurs and serves as a reference for future ecological monitoring, including post-GEM release assessments.

## Methods

### Study area

**Fig 1:**
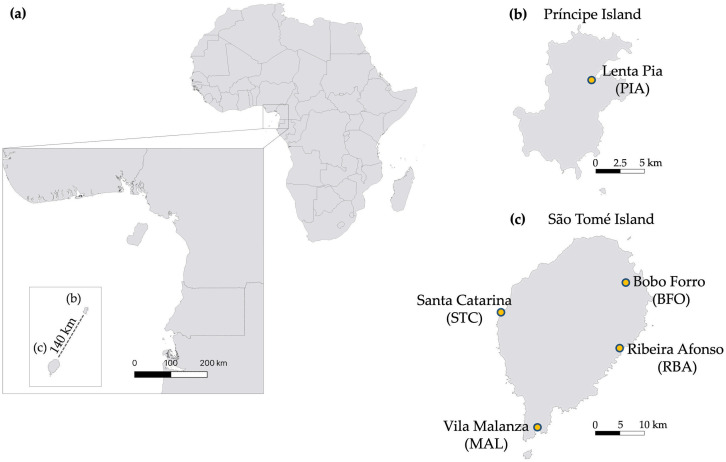
Geographic distribution of the study localities (yellow circles) on the islands of São Tomé and Príncipe. **(a)** Location of São Tomé and Príncipe in relation to the African continent; **(b)** Study locality on Príncipe Island; **(c)** Study localities on São Tomé Island. This map was created using the free and open-source software QGIS. Basemap and data from © OpenStreetMap contributors, available under the Open Database License (ODbL 1.0) (https://www.openstreetmap.org/copyright).

The study was performed on STP islands, located in the Gulf of Guinea, approximately 250 km off the coast of Gabon, Central Africa (Fig 1a). With total areas of 857 km^2^ and 139 km^2^, respectively, the islands are marked by mountain ranges and steep slopes, with flatter areas in the north and northeast. Both islands have numerous coastal streams and mangroves [8]. With an equatorial climate, the islands experience a short dry season (June to September) and an extended wet season (October to May).

A total of five localities were selected and sampled during the dry and wet seasons of 2022–2023. Four localities were on the main island, São Tomé, and one on the smaller island, Príncipe (Fig 1). The selected localities are all urban and known as larval habitats of *An. coluzzii*. A wide geographical spread of localities was included to capture spatial variation in aquatic community diversity. Within these selected habitats, we sampled habitats that differed in permanence and general physical features, not to compare specific larval habitat features, but to ensure that the survey encompassed the range of environments typically used by *An. coluzzii* on the islands. In São Tomé, collections were conducted in Santa Catarina (STC), Bobo Forro (BFO), Ribeira Afonso (RBA), and Vila Malanza (MAL). In Príncipe, collections were carried out in Lenta Pia (PIA) (Fig 1; S1 Table).

### Habitat identification and characterization

One permanent and one temporary larval habitat was identified for each area by a local entomologist from the National Center of Endemic Diseases (CNE) (S1 Fig). All larval habitats were screened before initiating the study to confirm the presence of *An. coluzzii* immature stages and only those habitats positive for *An. coluzzii* were selected. Permanent habitats were defined as larval habitats where water remained continuously or for a minimum of 3 months after the end of the wet season, allowing mosquitoes to reproduce throughout the year. Temporary habitats were defined as larval habitats where water remained for no more than approximately 1 month after the wet season ended. All selected larval habitats were georeferenced using a Garmin GPSMAP^®^ 64SX GPS device (Garmin, Olathe, USA).

Each habitat was characterized as one of the following larval habitat features: stream, ditch, swamp, roadside puddle (associated with compacted surfaces or depressions on the side of roads), small puddle, or artificial hole (used to store water during construction). Anthropogenic habitats were classified according to their well-defined boundaries and usage reports in construction, while natural habitats had irregular edges. Conditions such as water current, light intensity, turbidity, and the presence or absence of vegetation (inside the habitat) were visually estimated. The water current was visually characterized as slow, fluctuating, or still. Light intensity was visually categorized into light and shadow [14,15]. All visual classifications were performed by the same person (MJMC) to maintain consistency.

### Sampling aquatic biodiversity

Sampling was performed between May 2022 and June 2023. Collections were carried out twice in the dry season (September 2022 and June 2023) and twice in the wet season (May 2022 and January 2023) for a total of four sampling events per habitat over the course of the study. If the larval habitat was found dry, only the status, “dry”, was recorded.

For each water body, three edges were demarcated with 1 m flagging tape indicating each sweep. With a 30 cm D-frame kick net, one sweep was performed below the surface along the 1 m length of each of the three edges. The sample collected was poured into a tray with clean tap water and all organisms were collected with a plastic pipette and forceps. Organisms were stored in 5 ml tubes filled with 80% ethanol and transferred to the UCMI Molecular Biology Laboratory at the University of São Tomé and Príncipe (USTP) to be identified to the lowest taxonomic level possible.

Although taxonomic information exists for some groups of aquatic macroinvertebrates in STP [8], identification to species level is often limited by the availability of detailed, region-specific taxonomic keys and by the condition of collected material. Due to these constraints and to ensure consistency and comparability across samples, we adopted the family level as the standard taxonomic resolution, which was also the most common level of identification achieved in this study. The samples were then sent to the University of California, Davis, where identifications were confirmed. Morphological identification of macroinvertebrates was performed using taxonomic keys and scientific literature [24–29]. The identification of potential larval predators was carried out based on scientific literature [30,31]. Mosquito larvae were counted and identified to genus level (*Anopheles*, *Aedes* and *Culex*) using taxonomic keys [32–34]. The samples were subsequently curated following standard entomological procedures and deposited at the Bohart Museum of Entomology, University of California, Davis.

### Statistical analysis

All statistical analyses and plots drawn were performed using Rstudio software (R version 4.4.1) [35] and Microsoft Excel®. Data from both islands were combined due to differences in sample size (four localities on São Tomé Island and one locality on Príncipe Island). The following packages were used for the analysis: *vegan_2.6–8* [36], *car_3.1–3* [37], and *lmertest_3.1–3* [38].

Shannon-Wiener (*H’*), Simpson’s diversity (*D*), and Pielou’s equitability diversity (*J′*) indices were calculated using the following formulas [39]:

Shannon-Wiener Index (*H’*):


$$H^{\prime }=\;\sum pi\;\text{ln}\;pi,$$ (1)

Simpson*’*s Index of Diversity (*D*):


$$D=\;\text{1}-\frac{\sum n-(n-\text{1})}{N(N-\text{1})},$$ (2)

Pielou’s Equitability Diversity *(J’)*:


$$J^{\prime }=\frac{H^{\prime }}{\text{ln}S},$$ (3)

Distribution of the data was assessed using Shapiro-Wilk test, Q-Q plots and Levene’s tests. For each index, a Generalized Linear Mixed Model (GLMM) was used to determine how larval habitat type (permanent or temporary), season (wet or dry) and year (2022 or 2023) affected diversity. Two GLMMs were used to assess the variation in richness (number of families) and abundance (number of individuals) between larval habitat type, seasons and years. The data followed a Poisson distribution which was used as the distribution family in the model. For all models, habitat was included as a random effect to account for repeated measures due to resampling of the same habitats. All analyses were performed with taxonomic standardization at the family level.

To explore temporal (wet or dry season) patterns and differences between temporary and permanent larval habitats and years in macroinvertebrate assemblages, a Non-Metric Multidimensional Scaling (NMDS) analysis was performed. NMDS was used to visualize differences in community composition based on presence/absence data at family level, considering larval habitat type, season and year as group variables, helping to identify patterns of similarity across variables. A PERMANOVA analysis was performed using the *adonis2* function from the *vegan* package in R [36] to test the null hypothesis of no difference in assemblage structure across larval habitat type, season and years. Jaccard dissimilarity was used as the distance metric, with a maximum of 100 iterations to obtain the minimum stress value.

## Results

### Habitat identification and characterization

Ten larval habitats were identified and characterized, comprising two habitat types (one permanent and one temporary habitat) in each of the five localities (Table 1). Natural habitats predominated, with only one of the ten habitats being anthropogenic. Swamps were the most common larval habitat. All larval habitats were exposed to direct sunlight and most featured clear and still water. Natural vegetation was present in 70% of the habitats.

**Table 1:** Characteristics of *Anopheles coluzzii* larval habitats on the Islands of São Tomé and Príncipe during 2022–2023.

Locality	Larval habitat type	Nature of larval habitat	Larval habitat feature	Intensity of light	Turbidity	Vegetation	Water current
**STC**	permanent	natural	swamp	light	clear	present	still
	temporary	natural	stream	light	clear	absent	still
**BFO**	permanent	natural	small puddle	light	turbid	present	still
	temporary	natural	roadside puddle	light	clear	absent	still
**RBA**	permanent	man-made	artificial hole	light	clear	present	still
	temporary	natural	small puddle	light	turbid	present	still
**MAL**	permanent	natural	stream	light	clear	present	slow
	temporary	natural	swamp	light	turbid	present	still
**PIA**	permanent	natural	swamp	light	clear	present	still
	temporary	natural	roadside puddle	light	clear	absent	still

STC – Santa Catarina; BFO – Bobo Forro; RBA – Ribeira Afonso; MAL – Vila Malanza; PIA – Lenta Pia.

### Aquatic macroinvertebrates assemblages

**Fig 2:**
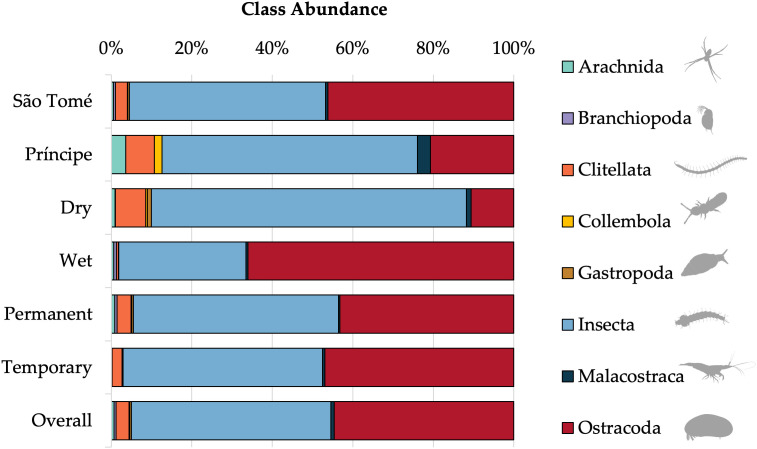
Class abundance of aquatic macroinvertebrates by island, season, larval habitat type, and overall. Abundance is shown for islands (São Tomé, Príncipe), seasons (dry, wet), and larval habitat types (permanent, temporary).

A total of 5,208 aquatic macroinvertebrates belonging to eight classes, 15 orders and 51 families were sampled across all localities in STP. Insecta was the predominant class with the highest diversity (49.6% of individuals, consisting of 30 families), Ostracoda was the next most abundant class (44.7% of individuals, in three families), followed by Clitellata (3.2% of individuals, in a single family) (Fig 2). Mosquito larvae of *Anopheles*, *Aedes*, and *Culex* were recorded in the sampled larval habitats. *Anopheles* larvae were found in both temporary and permanent habitats across all surveyed localities, although their abundance varied among habitats. A detailed summary of larval counts by genus, habitat type, and locality is provided in S2 Table.

### Assemblages according to habitat type and season

**Fig 3:**
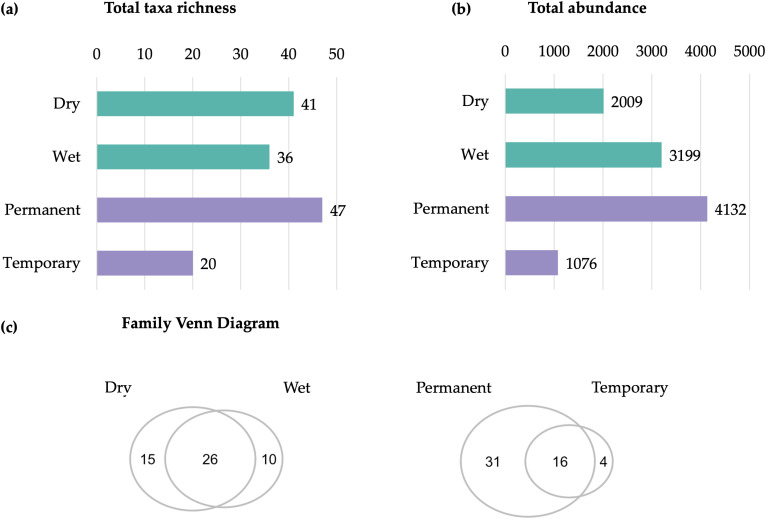
Family total taxa richness (a) and total abundance (b) of macroinvertebrates, and family-level Venn diagram (c) showing differences across sampling parameters. Sampling parameters include seasons (dry, wet) and habitat types (permanent, temporary).

Total abundance and family richness for larval habitat type and season are given in Fig 3. The family with the highest relative abundance in both permanent and temporary larval habitats was Cyprididae (Ostracoda: Podocopida) with 41.9% and 50.3%, respectively. This family was followed by Culicidae (Insecta: Diptera) in permanent habitats (33.1%), and Chironomidae (Insecta: Diptera) in temporary habitats (16.2%). In the wet season, the family Cyprididae (Ostracoda: Podocopida) had the highest relative abundance (66.0%), followed by Micronectidae (Insecta: Hemiptera) with 7.8%. In the dry season, the most abundant families were Culicidae (Insecta: Diptera) followed by Cyprididae (Ostracoda: Podocopida) with 65.6% and 7.9%, respectively. Some families were exclusively found in only one habitat type or season, which we summarize in Fig 3c (S3 Table).

**Fig 4:**
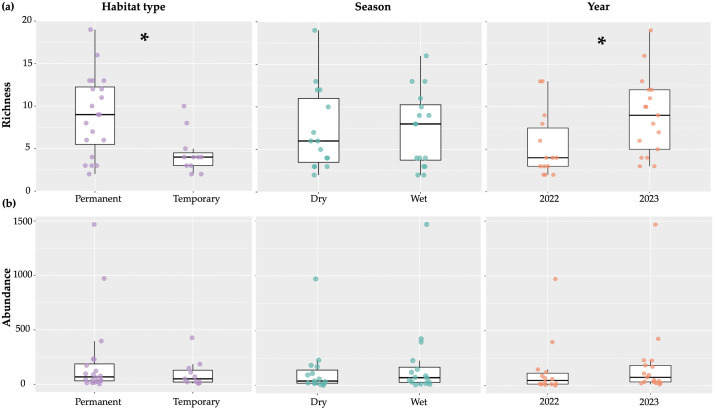
Richness (a) and Abundance (b) of aquatic macroinvertebrates across different habitat types and seasons. Asterisks (*) indicate significant differences (*p* < 0.05) between groups. The points represent the individual values of each sample, showing the dispersion of the data.

Overall, family richness was significantly higher in permanent compared to temporary habitats (Estimate = −4.51, z = −2.89, *p* = 0.01), while no significant differences were detected between the dry and wet seasons (Estimate = 0.36, z = 0.24, *p* = 0.81) (Fig 4a). Abundance did not significantly differ between permanent and temporary habitats (Estimate = −110.53, z = −1.02, *p* = 0.32) or seasons (Estimate = 71.66, z = 0.70, *p* = 0.49) (Fig 4b). As more collections were made on São Tomé (four localities) than Príncipe (one locality) we did not make statistical comparisons between the islands. Interannual variation analysis showed significantly higher richness in 2023 than in 2022 (Estimate = 0.60, z = 4.21, *p* < 0.001) (Fig 4a). However, total abundance did not differ significantly between years (Estimate = 0.66, z = 1.54, *p* = 0.125), indicating that increased richness was not accompanied by higher abundance (Fig 4b).

**Fig 5:**
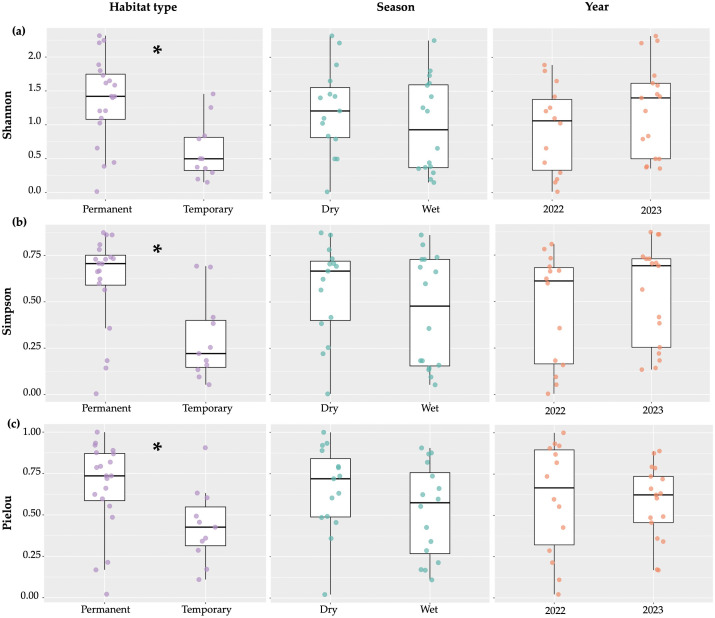
Shannon’s index (a), Simpson’s index (b) and Pielou’s index (c) of aquatic macroinvertebrate biodiversity across habitat types, seasons and years in São Tomé and Príncipe. Asterisks (*) indicate significant differences (*p* < 0.05) between groups. The points represent individual sample values, showing the dispersion of the data.

Shannon’s Index was significantly higher for permanent habitats indicating greater diversity (Estimate = −0.73, z = −3.66, *p* = 0.001), but no difference was found between seasons (Estimate = −0.20, z = −1.04, *p* = 0.31) or years (Estimate = 0.36, z = 1.87, *p* = 0.08) (Fig 5a). The mean Shannon’s Index for family diversity in permanent habitats was *H′* = 1.36, indicating moderate diversity, whereas temporary habitats presented a mean of *H′* = 0.61, suggesting low diversity of aquatic macroinvertebrates.

Simpson’s diversity index, where zero represents uniformity and 1 represents complete diversity, was significantly higher in permanent habitats (*D* = 0.61) than temporary habitats (*D* = 0.29) (Estimate = −0.31, z = −3.99, *p* = 0.001). There was no significant difference between seasons (Estimate = −0.10, z = −1.41, *p* = 0.17) or years (Estimate = 0.53, z = 1.77, *p* = 0.076) (Fig 5b).

Regarding evenness, Pielou’s index was significantly higher in permanent habitats (*J′* = 0.67) compared to temporary habitats (*J′* = 0.43), with higher values indicating more balanced distributions of family abundance (Fig 5c; Estimate = −0.23, z = −2.76, *p* = 0.01). There was no significant difference between seasons (Estimate = −0.12, z = −1.55, *p* = 0.13) or years (Estimate = −0.03, z = −0.38, *p* = 0.71) (Fig 5c). Since no significant differences were detected for seasonality or year across any of the indices, separate analyses were performed for permanent and temporary habitats. Nonetheless, the effect of seasonality remained non-significant (S2 Fig and S3 Fig).

**Fig 6:**
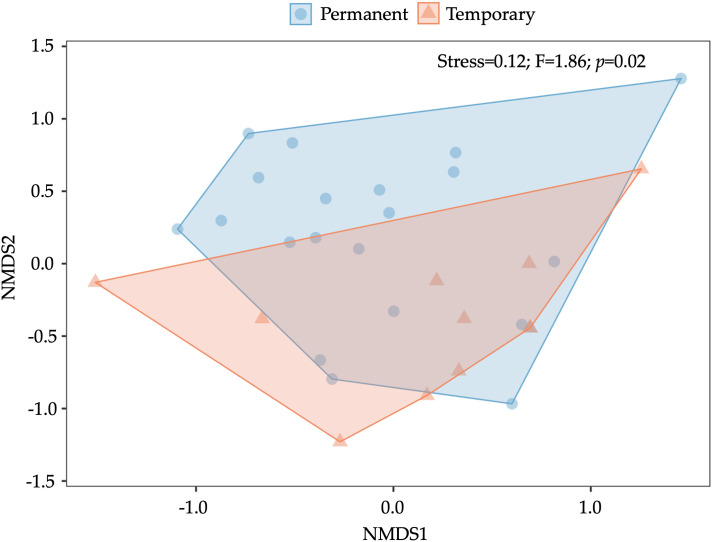
Non-metric multidimensional scaling (NMDS) plot based on a Jaccard similarity matrix, showing differences in aquatic macroinvertebrate composition between permanent and temporary habitats. Each point represents a sample, with colors and shapes indicating the habitat type (permanent or temporary). Polygons represent the convex hulls encompassing each group. 2D stress = 0.12.

Overall, non-metric multidimensional scaling (NMDS) did not show a significant effect of season (R^2^ = 0.05, F = 1.55, *p* = 0.07) and year (R^2^ = 0.04, F = 1.51, *p* = 0.07) on the aquatic macroinvertebrate composition (S4 Fig and S5 Fig). However, the results indicated that habitat type had a significant effect on composition (R^2^ = 0.06, F = 1.86, *p* = 0.02). Permanent and temporary habitat types were significantly different in terms of composition (based on Jaccard distance) (Fig 6).

### Predator identification

**Fig 7:**
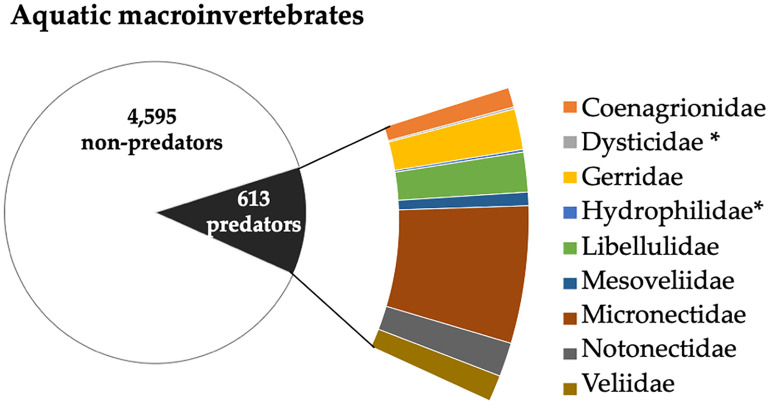
Proportion of potential predators of mosquito larvae in São Tomé and Príncipe islands and relative abundance of each identified predator family. Asterisks (*) indicate families with values below 1%.

Out of 5,208 macroinvertebrates sampled, 613 (11.7%) belonged to families harboring potential predators of mosquito larvae, including *An. coluzzii.* Nine families were represented, all from class Insecta (Fig 7). The most abundant family was Micronectidae, with 266 individuals (43.4%), followed by Gerridae (79 individuals; 12.9%) and Libellulidae (77 individuals; 12.6%).

## Discussion

Our study describes the aquatic macroinvertebrate community present in larval habitats typical of *An. coluzzii*, a member of the *Anopheles gambiae* complex and the primary malaria vector on the islands of STP. Permanent and temporary habitats were sampled over dry and wet seasons for two consecutive years to determine variation in biodiversity patterns in these aquatic ecosystems. The surveyed larval habitats hosted important mosquito disease vectors (*Anopheles*, *Aedes* and *Culex*), as well as a diverse macroinvertebrate community encompassing eight classes, 15 orders and 51 families. Insects made up half of the sample and were the most diverse class (30 families), followed by Ostracoda with 45% abundance, comprising three families. The remaining samples included small numbers of other crustaceans, spiders, annelid worms, springtails, and mollusks. Nine of the 51 families sampled are potential larval predators, which made up approximately 12% of the sample.

Members of the *Anopheles gambiae* complex are known for their ability to develop in a variety of larval habitats [15,40]. *Anopheles coluzzii*, the sole malaria vector on the islands of STP, has been reported to successfully develop in both temporary and permanent habitats, and natural and anthropogenic water sources, and our study is consistent with these earlier reports (S2 Table) [41,42]. Based on this known ecological range, our study included urban and coastal environments with a diversity of larval habitats typical of *An. coluzzii*. These features included swamps, streams, and puddles, with characteristics such as extensive sunlight exposure, presence of natural vegetation, and presence of debris (including organic matter and anthropogenic litter), as described by similar studies conducted in mainland West Africa [40,43].

Our study documented a diverse range of macroinvertebrates across larval habitats in STP. The most abundant of the 51 families were Cyprididae (Podocopida), Culicidae (Diptera), Chironomidae (Diptera), Micronectidae (Hemiptera), Naididae (Tubificida), Gerridae (Hemiptera), Libellulidae (Odonata), Baetidae (Ephemeroptera), and Notonectidae (Hemiptera) (S4 Table). Previous studies have shown that members of the orders Diptera, Odonata, Ephemeroptera, Hemiptera and Coleoptera, which include our most abundant families sampled, are commonly sampled together in freshwater habitats across mainland Africa [30,44–46].

We found increased macroinvertebrate diversity in permanent compared to temporary habitat types across all three diversity indices measured. These results were supported by the NMDS analysis, which showed differences in the structure of the communities depending on water permanence. Our findings highlight the role of stability in sustaining diverse communities. Permanent habitats are likely to host a greater diversity of species due to greater time for colonization of the habitat [4]. Conversely, frequent flooding and drying of temporary habitats results in more extreme environmental conditions, which may allow fewer taxa to survive (e.g., [47]). Other studies have found that temporary mosquito habitat type tends to support lower macroinvertebrate densities [30,46,48].

Temporary habitats are inherently more unpredictable, and the organisms inhabiting these environments often exhibit characteristics such as terrestrial adult stages or desiccation-resistance enabling them to survive when water is no longer available [49]. In contrast, permanent habitats tend to provide more stable and consistent conditions [50]. Our observations align with this pattern: families such as Chironomidae, Culicidae, and Cyprididae were the most abundant in temporary habitats (S4 Table). Dipteran groups typically have terrestrial adult forms and relatively short larval development times [51,52], whereas Cyprididae possess desiccation-resistant stages that enable survival through dry periods, with cysts hatching once water becomes available again [53]. Conversely, families such as Notonectidae, Coenagrionidae and Libellulidae showed higher abundance in permanent habitats (S4 Table). Adult Hemipterans are strong dispersers, consume a variety of prey types, and are often found in permanent habitats [4,54], whereas Odonates have long larval stages and depend on persistent water bodies for completed development [55].

It has been previously established that seasonality is an important factor shaping the structure of aquatic communities [56,57]. Li et al. [57] found a higher number of taxa at larval habitats during the dry season when compared to the wet season, attributing this pattern to the inability of some species to tolerate flooding and to greater abiotic variation. In regions with marked rainfall seasonality, heavy precipitation often increases water flow and substrate instability, causing drift and reducing the abundance and richness of aquatic communities [56,58]. However, in our study seasonal differences were non-significant. This lack of pronounced variation likely stems from the equatorial climate of STP, where the wet season extends for approximately eight months and the dry season lasts only four months [8,12]. This equatorial climate is characterized by less marked seasonal transitions, resulting in relatively stable conditions throughout the year. This likely mitigates the impact of seasonal variations on aquatic biodiversity [8].

Minor interannual variation was observed in aquatic macroinvertebrate communities, shown by richness estimates only, indicating a stable community composition between 2022 and 2023. Similarly, Guimarães et al. [59] reported little variation in richness throughout the year and concluded that stable seasonal patterns in tropical systems often support minimal temporal fluctuations in community structure. The persistence of similar taxonomic groups across years enhances the ability to detect changes attributable to vector management interventions [60,22]. This ecological stability underscores the suitability of these aquatic habitats and their macroinvertebrate communities for long-term ecological research and comprehensive risk assessments in GEM-based vector control strategies [10].

From a long-term monitoring perspective, future studies should aim to increase the number of larval habitats sampled to better represent the physical variability of these habitats. Expanding sampling coverage would also strengthen the identification of robust indicator taxa for longitudinal monitoring programs. Based on our findings and previous studies, the Libellulidae emerges as a promising candidate family, given its widespread occurrence in mosquito larval habitats, well-documented predatory roles, and previous use in long-term monitoring studies [61].

Potential larval predators made up 12% of the sample. All predators identified were insects, with families Micronectidae (Hemiptera), Gerridae (Hemiptera) and Libellulidae (Odonata) being the most abundant. Micronectidae are primarily described as herbivorous/detritivorous, but some species exhibit predatory behavior [31]. Studies have previously demonstrated their ability to feed on larval and pupal stages of *Aedes* under laboratory conditions [62–64]. As individuals in our study were not identified to species level and given the lack of local references on Micronectidae species occurring on the island, we considered this family as potential larval predators based on their documented feeding behavior. Other studies similarly identified Odonata and Hemiptera as important population regulators of mosquitoes [62,64]. These taxa are common components of tropical freshwater assemblages and contribute to maintaining ecosystem balance by regulating mosquito populations and other aquatic invertebrates [46].

Ouedraogo et al. [65] identified Gerridae and Libellulidae as potential larval predators, occurring in high abundances in larval habitats of *An. gambiae*. Aeshnidae, a family of Odonata, often found in typical *Anopheles* larval habitats on the mainland and known as efficient predators [30,65,66], were not recorded in our study. This likely reflects their low diversity and limited distribution in STP, with only two species described for the islands [8]. Historical records are scarce, suggesting very low abundance in STP. In contrast, Libellulidae were recorded in our study and are well-documented as present in typical larval habitats and as effective mosquito predators in western Africa [65]. On the African continent, Libellulidae exhibit relatively high species richness [67], which likely explains their wide distribution. Consistently, this family was among the most abundant potential predator families recorded in our study. Within the dragonfly assemblage, *Pantala flavescens*—reported by Ouedraogo et al. [65] in Burkina Faso—was the only species among the nine recorded in the study that also occurs on the STP islands [8]. Although members of the families Micronectidae and Gerridae were present in STP, no species-level records have been formally described for STP or other islands in the Gulf of Guinea, likely reflecting the limited taxonomic studies in the region.

Due to the limited availability of region-specific taxonomic keys for the macroinvertebrate groups sampled in the present study, specimens were identified only to the family level. A similar approach was used in previous studies on macroinvertebrate communities [30,46,68,69]. As in those studies, the diversity levels here described are likely to be underestimated and should thus be interpreted with caution. This is particularly relevant for families that include potential larval predators, for which further analyses would be valuable to identify the specific predator–prey relationships occurring in these habitats. On the other hand, the consistent trends observed between permanent and temporary habitat types are less likely to be biased by the taxonomic resolution, especially considering that island ecosystems usually support lower overall species diversity [70].

## Conclusions

Our study provides the first detailed characterization of aquatic macroinvertebrate diversity in mosquito larval habitats in STP, documenting a wide range of taxa including insects, crustaceans, spiders, annelid worms, springtails, and mollusks at the family level. Diversity was higher in permanent as opposed to temporary habitats, reinforcing the importance of habitat stability for maintaining diverse communities. The insular context of STP, characterized by limited colonization and lower species richness compared to continental systems, may simplify ecological interactions but also emphasizes the importance of understanding these dynamics for monitoring and conserving biodiversity. Our findings emphasize the need for comprehensive taxonomic studies to accurately characterize aquatic communities providing a baseline for future research. Overall, these findings reveal the ecological complexity of larval habitats and support the incorporation of community-level perspectives to advance tropical freshwater ecology and inform future studies on ecological interactions.

## Supporting information

S1 Fig Sampling sites selected in each locality on the study. A) STC permanent; B) STC temporary; C) BFO permanent; D) BFO temporary; E) RBA permanent; F) RBA temporary; G) MAL permanent; H) MAL temporary; I) PIA permanent; J) PIA temporary.
(TIF)

S2 Fig Richness (a) and Abundance (b) of aquatic macroinvertebrates across seasons, analyzed separately for temporary and permanent sites. Asterisks (*) indicate significant differences (p < 0.05) between groups. The points represent the individual values of each sample, showing the dispersion of the data.
(TIF)

S3 Fig Shannon’s index (a), Simpson’s index (b) and Pielou’s index (c) of aquatic macroinvertebrate biodiversity across seasons, analyzed separately for temporary and permanent sites. Asterisks (*) indicate significant differences (p < 0.05) between groups. The points represent individual sample values, showing the dispersion of the data.
(TIF)

S4 Fig Non-metric multidimensional scaling (NMDS) plot based on a Jaccard similarity matrix, showing aquatic macroinvertebrate composition during the wet and dry seasons. No significant differences were detected between seasons. 2D stress = 0.12.
(TIF)

S5 Fig Non-metric multidimensional scaling (NMDS) plot based on a Jaccard similarity matrix, showing aquatic macroinvertebrate composition across sampling years. No significant differences were observed between years. 2D stress = 0.12.
(TIF)

S1 Table Geographic coordinates of larval habitats by locality on the Islands of São Tomé and Príncipe. (DOCX)

S2 Table Abundance of mosquito larvae collected in larval habitats of *Anopheles* across localities and habitat types. (DOCX)

S3 Table Presence and absence of aquatic macroinvertebrates collected on different sampling seasons and habitat types on the Islands of São Tomé and Príncipe. (DOCX)

S4 Table The abundance (A) and relative abundance (%RA) of macroinvertebrates in different sampling seasons and habitat type on the islands of São Tomé and Príncipe. (DOCX)
