# Supplementary figures and images for: Aquatic macroinvertebrate diversity in mosquito larval habitats in São Tomé and Príncipe
Source: PLoS One. 2026 Jan 6;21(1):e0339486. doi: 10.1371/journal.pone.0339486 (PMC12774360; doi:10.1371/journal.pone.0339486)

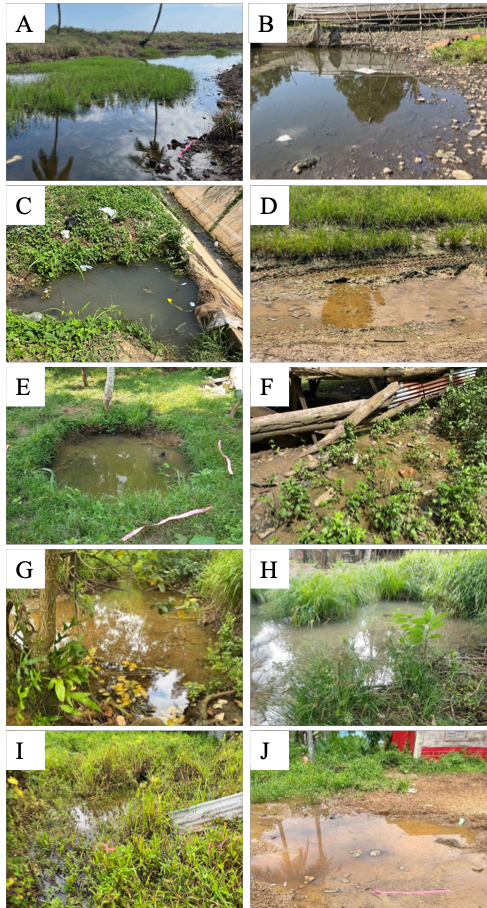

Supplement: S1 Fig — A) STC permanent; B) STC temporary; C) BFO permanent; D) BFO temporary; E) RBA permanent; F) RBA temporary; G) MAL permanent; H) MAL temporary; I) PIA permanent; J) PIA temporary. (TIF) [file pone.0339486.s001.tif]

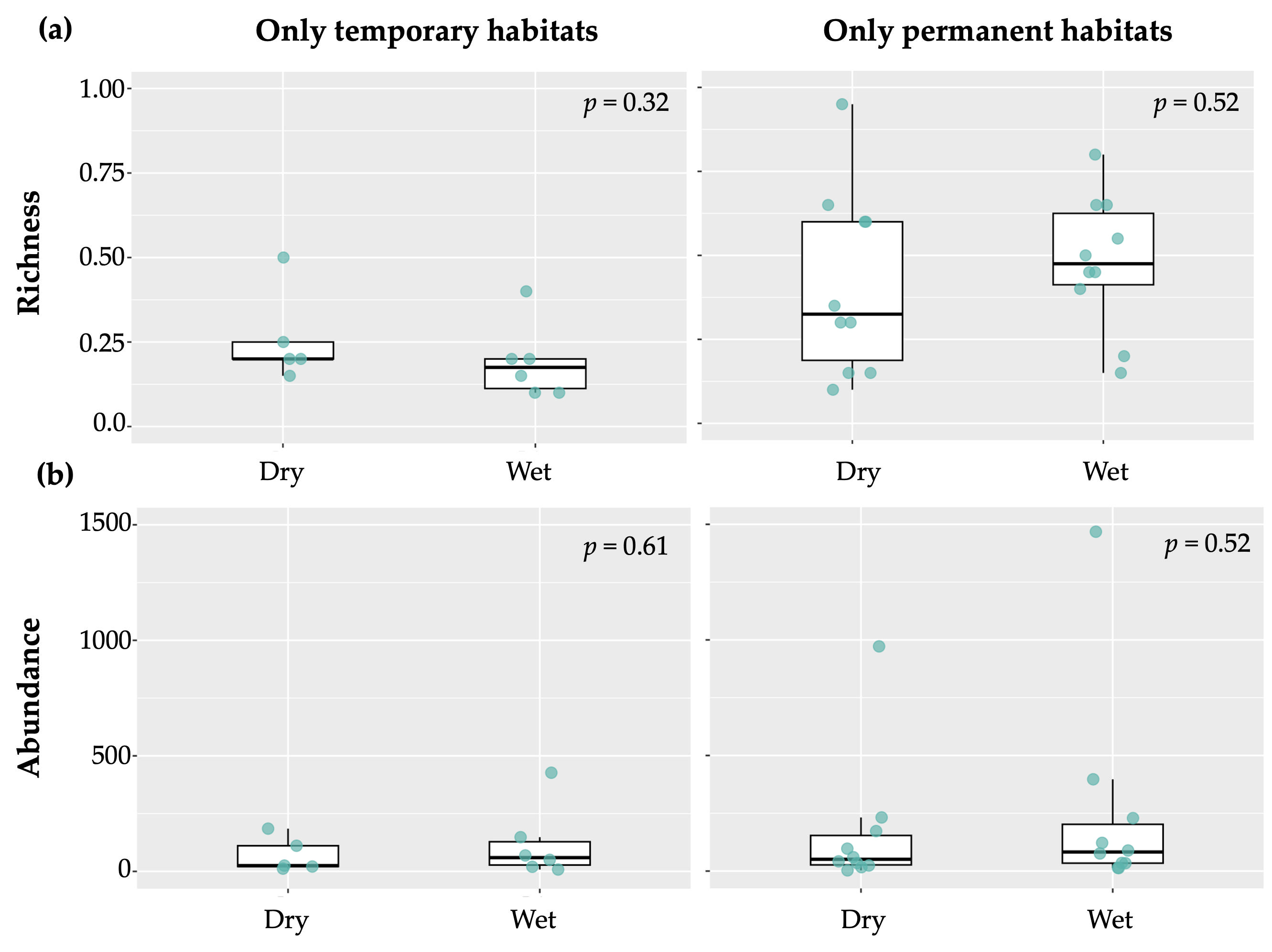

Supplement: S2 Fig — Asterisks (*) indicate significant differences (p < 0.05) between groups. The points represent the individual values of each sample, showing the dispersion of the data. (TIF) [file pone.0339486.s002.tif]

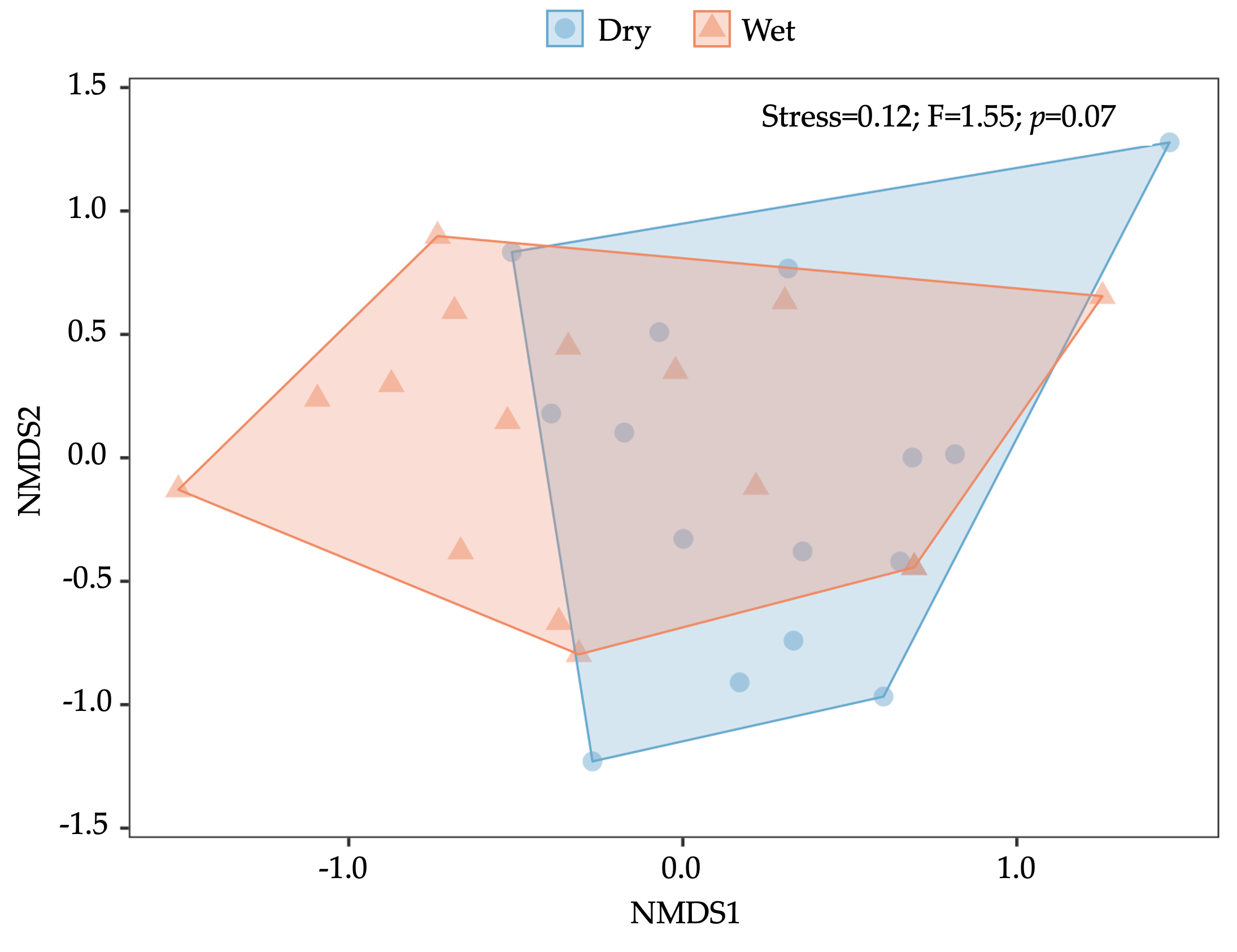

Supplement: S4 Fig — No significant differences were detected between seasons. 2D stress = 0.12. (TIF) [file pone.0339486.s004.tif]

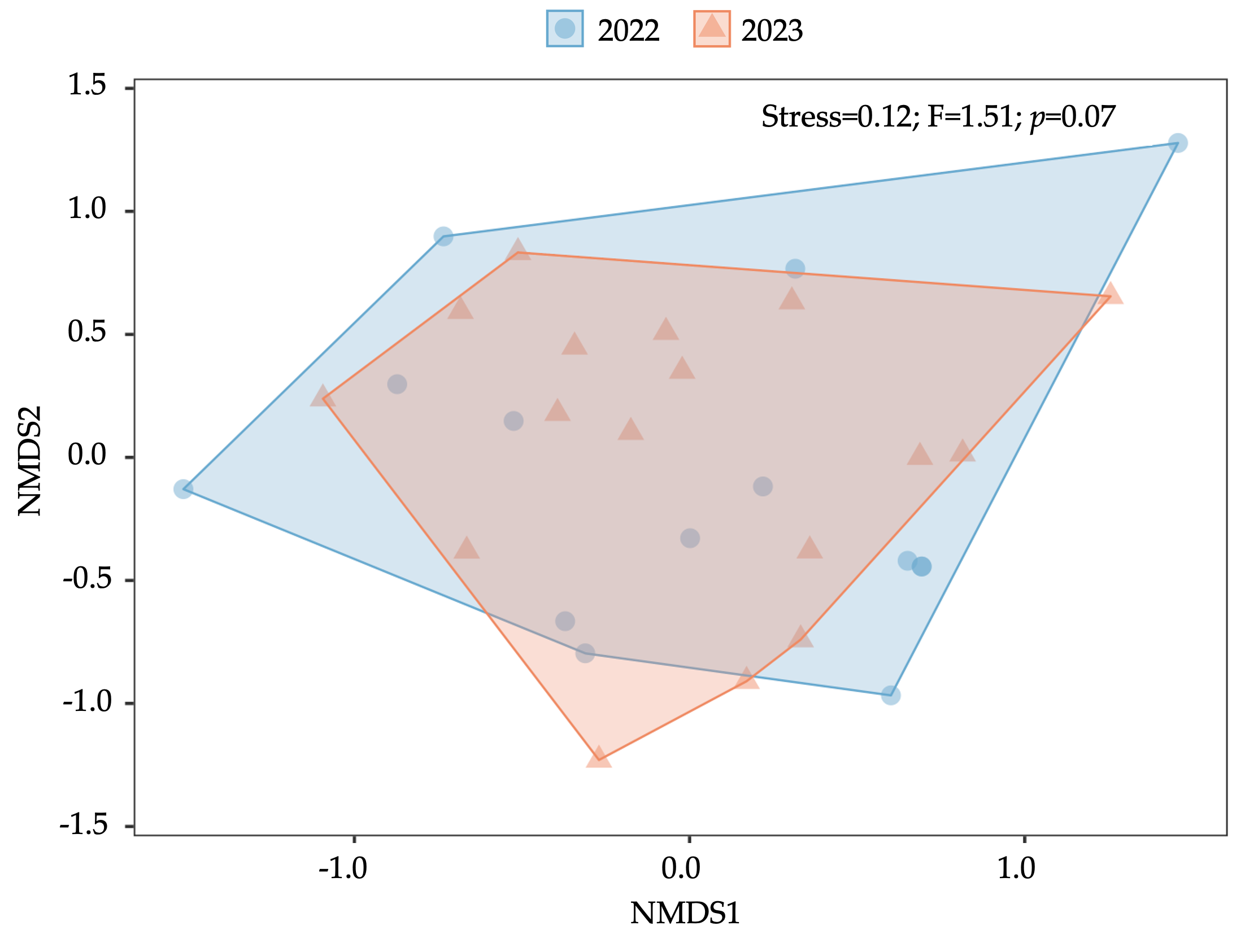

Supplement: S5 Fig — No significant differences were observed between years. 2D stress = 0.12. (TIF) [file pone.0339486.s005.tif]
