# Supplementary material for: Aquatic macroinvertebrate diversity in mosquito larval habitats in São Tomé and Príncipe
Source: PLoS One. 2026 Jan 6;21(1):e0339486. doi: 10.1371/journal.pone.0339486 (PMC12774360; doi:10.1371/journal.pone.0339486)
Supplement: S1 Table — (DOCX) [file pone.0339486.s006.docx]

**S1 Table.** Geographic coordinates of larval habitats by locality on the Islands of São Tomé and Príncipe.

| **Island** | **Locality** | **Habitat type** | **Latitude** | **Longitude** |
| --- | --- | --- | --- | --- |
| São Tomé | Santa Catarina (STC) | Permanent | 0°16'01.0"N | 6°28'10.0"E |
|  |  | Temporary | 0°16'14.7"N | 6°28'31.7"E |
|  | Bobo Forro (BFO) | Permanent | 0°19'27.4"N | 6°42'24.7"E |
|  |  | Temporary | 0°19'30.9"N | 6°42'22.7"E |
|  | Vila Malanza (MAL) | Permanent | 0°02'55.9"N | 6°32'12.8"E |
|  |  | Temporary | 0°02'54.2"N | 6°32'11.2"E |
|  | Ribeira Afonso (RBA) | Permanent | 0°11'48.2"N | 6°41'57.3"E |
|  |  | Temporary | 0°11'48.3"N | 6°41'57.3"E |
| Príncipe | Lenta Pia (PIA) | Permanent | 1°38'17.7"N | 7°25'03.9"E |
|  |  | Temporary | 1°38'15.5"N | 7°24'59.5"E |
