# Supplementary material for: Aquatic macroinvertebrate diversity in mosquito larval habitats in São Tomé and Príncipe
Source: PLoS One. 2026 Jan 6;21(1):e0339486. doi: 10.1371/journal.pone.0339486 (PMC12774360; doi:10.1371/journal.pone.0339486)
Supplement: S2 Table — (DOCX) [file pone.0339486.s007.docx]

**S2 Table.** Abundance of mosquito larvae collected in larval habitats of *Anopheles* across localities and habitat types.

| Island | Locality | Habitat type | *Anopheles* | *Aedes* | *Culex* | Total |
| --- | --- | --- | --- | --- | --- | --- |
| São Tomé | Santa Catarina (STC) | Temporary | 1 | 0 | 0 | 1 |
|  |  | Permanent | 1 | 0 | 970 | 971 |
|  | Bobo Forro (BFO) | Temporary | 35 | 0 | 14 | 49 |
|  |  | Permanent | 45 | 6 | 22 | 73 |
|  | Vila Malanza (MAL) | Temporary | 32 | 0 | 5 | 37 |
|  |  | Permanent | 18 | 0 | 5 | 23 |
|  | Ribeira Afonso (RBA) | Temporary | 18 | 0 | 1 | 19 |
|  |  | Permanent | 217 | 0 | 22 | 239 |
| Príncipe | Lenta Pia (PIA) | Temporary | 8 | 0 | 1 | 9 |
|  |  | Permanent | 19 | 17 | 13 | 49 |
| Total |  |  | 394 | 23 | 1053 | 1470 |
