# Supplementary material for: Aquatic macroinvertebrate diversity in mosquito larval habitats in São Tomé and Príncipe
Source: PLoS One. 2026 Jan 6;21(1):e0339486. doi: 10.1371/journal.pone.0339486 (PMC12774360; doi:10.1371/journal.pone.0339486)
Supplement: S3 Table — (DOCX) [file pone.0339486.s008.docx]

**S3 Table.** Presence and absence of aquatic macroinvertebrates collected on different sampling seasons and habitat types on the Islands of São Tomé and Príncipe.

| **Island** | **Family** | **Dry** | **Wet** | **Temporary** | **Permanent** |
| --- | --- | --- | --- | --- | --- |
| São Tomé | Agaonidae^a^ | + | - | - | + |
|  | Ampullariidae^a^ | + | - | - | + |
|  | Anapidae | + | + | - | + |
|  | Aphididae | - | + | - | + |
|  | Apidae | - | + | - | + |
|  | Araneidae^a^ | - | + | - | + |
|  | Baetidae | + | + | + | + |
|  | Cecidomyiidae^a^ | - | + | - | + |
|  | Ceratopogonidae | + | + | + | + |
|  | Chalcidoidea^a^ | - | + | - | + |
|  | Chironomidae | + | + | + | + |
|  | Chloropidae^a^ | + | - | - | + |
|  | Chrysomelidae^a^ | - | + | - | + |
|  | Coenagrionidae | - | + | + | + |
|  | Culicidae | + | + | + | + |
|  | Cyprididae | + | + | + | + |
|  | Daphniidae^a^ | + | - | + | - |
|  | Delphacidae^a^ | - | + | - | + |
|  | Diapriidae | + | - | - | + |
|  | Dolichopodidae^a^ | + | - | + | - |
|  | Drosophilidae | + | - | - | + |
|  | Dysticidae | - | + | - | + |
|  | Ephydridae^a^ | + | + | + | + |
|  | Formicidae | + | + | + | + |
|  | Gerridae | + | + | + | + |
|  | Hemipsocidae^a^ | - | + | - | + |
|  | Hydrophilidae^a^ | + | + | - | + |
|  | Isotomidae | + | + | + | + |
|  | Libellulidae | + | + | + | + |
|  | Linyphiidae | - | + | - | + |
|  | Lycosidae | + | + | - | + |
|  | Mesoveliidae^a^ | + | + | - | + |
|  | Micronectidae | + | + | + | + |
|  | Mysmenidae^a^ | + | - | + | - |
|  | Naididae | + | + | + | + |
|  | Notodromadidae | + | - | + | + |
|  | Notonectidae^a^ | + | + | - | + |
|  | Oxycarenidae^a^ | - | + | - | + |
|  | Palaemonidae | + | + | + | + |
|  | Pisauridae | + | - | - | + |
|  | Psychodidae^a^ | + | + | + | + |
|  | Saldidae^a^ | + | - | + | - |
|  | Salticidae | + | - | - | + |
|  | Tetragnathidae | + | + | - | + |
|  | Tetrigidae^a^ | + | - | - | + |
|  | Theridiidae^a^ | - | + | - | + |
|  | Veliidae | + | + | - | + |
| Príncipe | Anapidae | + | - | - | + |
|  | Aphididae | + | - | - | + |
|  | Apidae | + | - | - | + |
|  | Atyidae^b^ | - | + | - | + |
|  | Baetidae | - | + | - | + |
|  | Ceratopogonidae | + | + | - | + |
|  | Chironomidae | + | + | - | + |
|  | Coenagrionidae | + | + | - | + |
|  | Culicidae | + | + | + | + |
|  | Cyprididae | + | - | + | + |
|  | Diapriidae | - | + | - | + |
|  | Drosophilidae | + | - | - | + |
|  | Dysticidae | + | - | - | + |
|  | Formicidae | + | + | - | + |
|  | Gerridae | + | + | - | + |
|  | Isotomidae | + | + | + | + |
|  | Libellulidae | + | + | - | + |
|  | Limnocytheridae^b^ | + | - | - | + |
|  | Linyphiiidae | - | + | - | + |
|  | Lycosidae | + | - | - | + |
|  | Meenoplidae^b^ | + | - | - | + |
|  | Micronectidae | + | - | - | + |
|  | Naididae | + | - | + | + |
|  | Notodromadidae | + | - | - | + |
|  | Palaemonidae | - | + | - | + |
|  | Pisauridae | + | - | - | + |
|  | Salticidae | + | - | - | + |
|  | Tetragnathidae | + | - | - | + |
|  | Trombidiidae^b^ | + | - | - | + |
|  | Veliidae | + | + | - | + |

^a^ Families exclusive to São Tomé Island

^b^ Families exclusive to Príncipe Island
