# Supplementary material for: Aquatic macroinvertebrate diversity in mosquito larval habitats in São Tomé and Príncipe
Source: PLoS One. 2026 Jan 6;21(1):e0339486. doi: 10.1371/journal.pone.0339486 (PMC12774360; doi:10.1371/journal.pone.0339486)
Supplement: S4 Table — (DOCX) [file pone.0339486.s009.docx]

**S4 Table.** The abundance (A) and relative abundance (%RA) of macroinvertebrates in different sampling seasons and habitat type on the islands of São Tomé and Príncipe.

| **Islands** | **Family** | **Dry** | | **Wet** | | **Temporary** | | **Permanent** | | **Total of sample** | | |  |
| --- | --- | --- | --- | --- | --- | --- | --- | --- | --- | --- | --- | --- | --- |
|  |  | **A** | **%RA** | **A** | **%RA** | **A** | **%RA** | **A** | **%RA** | | **A** | **%RA** | |
| São Tomé and Príncipe | Agaonidae^a^ | 1 | 0,05 | 0 | 0,00 | 0 | 0,00 | 1 | 0,02 | | 1 | 0,02 | |
|  | Ampullariidae^a^ | 19 | 0,95 | 0 | 0,00 | 0 | 0,00 | 19 | 0,46 | | 19 | 0,36 | |
|  | Anapidae | 3 | 0,15 | 3 | 0,09 | 0 | 0,00 | 6 | 0,15 | | 6 | 0,12 | |
|  | Aphididae | 1 | 0,05 | 2 | 0,06 | 0 | 0,00 | 3 | 0,07 | | 3 | 0,06 | |
|  | Apidae | 1 | 0,05 | 1 | 0,03 | 0 | 0,00 | 2 | 0,05 | | 2 | 0,04 | |
|  | Araneidae^a^ | 0 | 0,00 | 1 | 0,03 | 0 | 0,00 | 1 | 0,02 | | 1 | 0,02 | |
|  | Atyidae^b^ | 0 | 0,00 | 4 | 0,13 | 0 | 0,00 | 4 | 0,10 | | 4 | 0,08 | |
|  | Baetidae | 18 | 0,90 | 52 | 1,63 | 8 | 0,74 | 62 | 1,50 | | 70 | 1,34 | |
|  | Cecidomyiidae^a^ | 0 | 0,00 | 1 | 0,03 | 0 | 0,00 | 1 | 0,02 | | 1 | 0,02 | |
|  | Ceratopogonidae | 19 | 0,95 | 6 | 0,19 | 14 | 1,30 | 11 | 0,27 | | 25 | 0,48 | |
|  | Chalcidoidea^a^ | 0 | 0,00 | 1 | 0,03 | 0 | 0,00 | 1 | 0,02 | | 1 | 0,02 | |
|  | Chironomidae | 89 | 4,43 | 246 | 7,69 | 174 | 16,17 | 161 | 3,90 | | 335 | 6,43 | |
|  | Chloropidae^a^ | 1 | 0,05 | 0 | 0,00 | 0 | 0,00 | 1 | 0,02 | | 1 | 0,02 | |
|  | Chrysomelidae^a^ | 0 | 0,00 | 1 | 0,03 | 0 | 0,00 | 1 | 0,02 | | 1 | 0,02 | |
|  | Coenagrionidae | 11 | 0,55 | 28 | 0,88 | 2 | 0,19 | 37 | 0,90 | | 39 | 0,75 | |
|  | Culicidae | 1317 | 65,56 | 168 | 5,25 | 116 | 10,78 | 1369 | 33,13 | | 1485 | 28,51 | |
|  | Cyprididae | 159 | 7,91 | 2113 | 66,05 | 541 | 50,28 | 1731 | 41,89 | | 2272 | 43,63 | |
|  | Daphniidae^a^ | 1 | 0,05 | 0 | 0,00 | 1 | 0,09 | 0 | 0,00 | | 1 | 0,02 | |
|  | Delphacidae^a^ | 0 | 0,00 | 23 | 0,72 | 0 | 0,00 | 23 | 0,56 | | 23 | 0,44 | |
|  | Diapriidae | 1 | 0,05 | 1 | 0,03 | 0 | 0,00 | 2 | 0,05 | | 2 | 0,04 | |
|  | Dolichiopodidae^a^ | 1 | 0,05 | 0 | 0,00 | 1 | 0,09 | 0 | 0,00 | | 1 | 0,02 | |
|  | Drosophilidae | 2 | 0,10 | 0 | 0,00 | 0 | 0,00 | 2 | 0,05 | | 2 | 0,04 | |
|  | Dysticidae | 2 | 0,10 | 2 | 0,06 | 0 | 0,00 | 4 | 0,10 | | 4 | 0,08 | |
|  | Ephydridae^a^ | 9 | 0,45 | 1 | 0,03 | 6 | 0,56 | 4 | 0,10 | | 10 | 0,19 | |
|  | Formicidae | 11 | 0,55 | 10 | 0,31 | 4 | 0,37 | 17 | 0,41 | | 21 | 0,40 | |
|  | Gerridae | 7 | 0,35 | 72 | 2,25 | 1 | 0,09 | 78 | 1,89 | | 79 | 1,52 | |
|  | Hemipsocidae^a^ | 0 | 0,00 | 1 | 0,03 | 0 | 0,00 | 1 | 0,02 | | 1 | 0,02 | |
|  | Hydrophilidae^a^ | 2 | 0,10 | 3 | 0,09 | 0 | 0,00 | 5 | 0,12 | | 5 | 0,10 | |
|  | Isotomidae | 8 | 0,40 | 2 | 0,06 | 3 | 0,28 | 7 | 0,17 | | 10 | 0,19 | |
|  | Libellulidae | 40 | 1,99 | 37 | 1,16 | 2 | 0,19 | 75 | 1,82 | | 77 | 1,48 | |
|  | Limnocytheridae ^b^ | 21 | 1,05 | 0 | 0,00 | 0 | 0,00 | 21 | 0,51 | | 21 | 0,40 | |
|  | Linyphiiidae | 0 | 0,00 | 2 | 0,06 | 0 | 0,00 | 2 | 0,05 | | 2 | 0,04 | |
|  | Lycosidae | 2 | 0,10 | 4 | 0,13 | 0 | 0,00 | 6 | 0,15 | | 6 | 0,12 | |
|  | Meenoplidae^b^ | 1 | 0,05 | 0 | 0,00 | 0 | 0,00 | 1 | 0,02 | | 1 | 0,02 | |
|  | Mesoveliidae^a^ | 2 | 0,10 | 24 | 0,75 | 0 | 0,00 | 26 | 0,63 | | 26 | 0,50 | |
|  | Micronectidae | 15 | 0,75 | 251 | 7,85 | 149 | 13,85 | 117 | 2,83 | | 266 | 5,11 | |
|  | Mysmenidae^a^ | 1 | 0,05 | 0 | 0,00 | 1 | 0,09 | 0 | 0,00 | | 1 | 0,02 | |
|  | Naididae | 152 | 7,57 | 16 | 0,50 | 26 | 2,42 | 142 | 3,44 | | 168 | 3,23 | |
|  | Notodromadidae | 33 | 1,64 | 0 | 0,00 | 1 | 0,09 | 32 | 0,77 | | 33 | 0,63 | |
|  | Notonectidae^a^ | 4 | 0,20 | 62 | 1,94 | 0 | 0,00 | 66 | 1,60 | | 66 | 1,27 | |
|  | Oxycarenidae^a^ | 0 | 0,00 | 1 | 0,03 | 0 | 0,00 | 1 | 0,02 | | 1 | 0,02 | |
|  | Palaemonidae | 24 | 1,19 | 11 | 0,34 | 24 | 2,23 | 11 | 0,27 | | 35 | 0,67 | |
|  | Pisauridae | 2 | 0,10 | 5 | 0,16 | 0 | 0,00 | 2 | 0,05 | | 2 | 0,04 | |
|  | Psychodidae^a^ | 2 | 0,10 | 0 | 0,00 | 1 | 0,09 | 6 | 0,15 | | 7 | 0,13 | |
|  | Saldidae^a^ | 1 | 0,05 | 0 | 0,00 | 1 | 0,09 | 0 | 0,00 | | 1 | 0,02 | |
|  | Salticidae | 4 | 0,20 | 0 | 0,00 | 0 | 0,00 | 4 | 0,10 | | 4 | 0,08 | |
|  | Tetragnathidae | 5 | 0,25 | 7 | 0,22 | 0 | 0,00 | 12 | 0,29 | | 12 | 0,23 | |
|  | Tetrigidae^a^ | 1 | 0,05 | 0 | 0,00 | 0 | 0,00 | 1 | 0,02 | | 1 | 0,02 | |
|  | Theridiidae^a^ | 0 | 0,00 | 1 | 0,03 | 0 | 0,00 | 1 | 0,02 | | 1 | 0,02 | |
|  | Trombidiidae^b^ | 1 | 0,05 | 0 | 0,00 | 0 | 0,00 | 1 | 0,02 | | 1 | 0,02 | |
|  | Veliidae | 15 | 0,75 | 36 | 1,13 | 0 | 0,00 | 51 | 1,23 | | 51 | 0,98 | |
| Total |  | 2009 | 100,00 | 3199 | 100,00 | 1076 | 100,00 | 4132 | 100,00 | | 5208 | 100,00 | |

^a^ Families exclusive to São Tomé Island

^b^ Families exclusive to Príncipe Island
